# A convention-radiomics CT nomogram for differentiating fat-poor angiomyolipoma from clear cell renal cell carcinoma

**DOI:** 10.1038/s41598-021-84244-3

**Published:** 2021-02-25

**Authors:** Yanqing Ma, Weijun Ma, Xiren Xu, Zheng Guan, Peipei Pang

**Affiliations:** 1 Zhejiang Provincial People’s Hospital, People’s Hospital of Hangzhou Medical College, Hangzhou, 310000 China; 2 Shaoxing City Keqiao District Hospital of Traditional Chinese Medicine, Shaoxing, 312000 China; 3 GE Healthcare, Hangzhou, 310000 China

**Keywords:** Cancer imaging, Renal cell carcinoma

## Abstract

This study aimed to construct convention-radiomics CT nomogram containing conventional CT characteristics and radiomics signature for distinguishing fat-poor angiomyolipoma (fp-AML) from clear-cell renal cell carcinoma (ccRCC). 29 fp-AML and 110 ccRCC patients were enrolled and underwent CT examinations in this study. The radiomics-only logistic model was constructed with selected radiomics features by the analysis of variance (ANOVA)/Mann–Whitney (MW), correlation analysis, and Least Absolute Shrinkage and Selection Operator (LASSO), and the radiomics score (rad-score) was computed. The convention-radiomics logistic model based on independent conventional CT risk factors and rad-score was constructed for differentiating. Then the relevant nomogram was developed. Receiver operation characteristic (ROC) curves were calculated to quantify the accuracy for distinguishing. The rad-score of ccRCC was smaller than that of fp-AML. The convention-radioimics logistic model was constructed containing variables of enhancement pattern, V_UP_, and rad-score. To the entire cohort, the area under the curve (AUC) of convention-radiomics model (0.968 [95% CI 0.923–0.990]) was higher than that of radiomics-only model (0.958 [95% CI 0.910–0.985]). Our study indicated that convention-radiomics CT nomogram including conventional CT risk factors and radiomics signature exhibited better performance in distinguishing fp-AML from ccRCC.

## Introduction

Renal angiomyolipoma (AML) is the most common benign renal neoplasm^1^, which derived from perivascular epithelial cells^2^ and composed of differing proportions of dysmorphic blood vessels, smooth muscle cells, and mature adipose fat^3^. The detection of macroscopic fat is reliable to establish the diagnosis as AML^4^. However, approximately 5% of AMLs contain undetectable fat^5^, which are termed as fp-AML. Jinzaki et al. categorized these triphasic tumors into classic and fp-AML subtype^6^. Renal cell carcinoma (RCC) is the ninth most common cancer globally^7^, accounting for approximately 3.8% of new cancers^8^. Moreover, ccRCC is the most common and aggressive subtype of RCC^8^. CcRCC has a worse prognosis and accounts for 94% of metastatic RCC^9^. There are some visual similarities between fp-AML and ccRCC. About 60% of fp-AMLs displayed hyperattenuating on unenhanced CT^6^. However, a proportion of ccRCCs showed similar hyperattenuating, and it had been summarized that no accurate threshold of CT attenuating could differentiate fp-AML from ccRCC^4^. RCC frequently contains macroscopic fat because of perinephric fat engulfment, lipid-producing necrosis, or osseous metaplasia^10^. About 60% of ccRCCs are observed cytoplasmic lipid^11^. The early avid and washout over time enhancement pattern does not help to distinguish fp-AML from ccRCC^12^. The overlapping imaging features and atypical findings limit the preoperative distinction between fp-AML and ccRCC, which lead to unnecessary surgeries on benign neoplasms^13^. The entire difference in prognosis and treatment of ccRCC and fp-AML makes the diagnosis of great clinical significance.

Radiomics is an emerging method, which extracts large amounts of objective, quantitative data from images. Radiomics features relate to histogram parameters, texture parameters, form factor parameters, gray-level co-occurrence matrix (GLCM) parameters, and gray-level run-length matrix (RLM) parameters. It has been used to predict the Fuhrman grade of RCC by CT-based radiomics features^8^. Previous studies have demonstrated that radiomics analysis has high accuracy in differentiating benign and malignant renal tumors^14^. CcRCC tends to achieve more inhomogeneous texture^15^ and shows a more rounded appearance shape^16^, compared with fp-AML. While the histogram analysis of attenuation measurement cannot distinguish fp-AML from RCC with 100% specificity^17^. Therefore, radiomics-only analysis is not sufficient for diagnosis making. The radiomics signature associated with conventional CT analysis has become a major trend.

To best of our knowledge, there has been no convention-radiomics CT nomogram study in distinguishing fp-AML from ccRCC. In this study, we quantified the radiomics signature by rad-score and sought to construct a convention-radiomics CT nomogram that covering both rad-score and conventional CT characteristics for better differentiating two diseases.

## Results

### General information and conventional CT analysis

There were 139 patients enrolled, 29 fp-AML patients (58.6% female, mean age 47.3 ± 10.9 years) and 110 ccRCC patients (30.0% female, mean age 59.5 ± 12.0 years). There was no statistic significance in variables of position (*p* = 0.277), calcification (*p* = 0.660), V_CMP_ (*p* = 0.115), and V_NP_ (*p* = 0.063). There was statistic significance in variables of cyst (*p* < 0.001), pseudocapsule (*p* < 0.001), enhancement pattern (*p* < 0.001), V_UP_ (*p* < 0.001), R_CMP_% (*p* < 0.001), and R_NP_% (*p* = 0.034) (Table 1).

**Table 1:** Conventional CT analysis.

Conventional CT characteristics	*p*
Position	0.277
Pseudocapsule	< 0.001
Calcification	0.660
Cyst	< 0.001
Enhancement pattern	< 0.001
V_UP_	< 0.001
V_CMP_	0.115
V_NP_	0.063
R_CMP_%	< 0.001
R_NP_%	0.034

The conventional qualitative CT characteristics including position, pseudocapsule, calcification, cyst, enhancement pattern were compared with the method of Pearson chi-square test. And the conventional quantitative CT characteristics including V_UP_, V_CMP_, V_NP_, R_CMP_%, and R_NP_% were compared with the method of independent-sample T-test. A *p* < 0.05 showed significant difference.

### Construction of radiomics signature and accuracy

**Figure 1:**
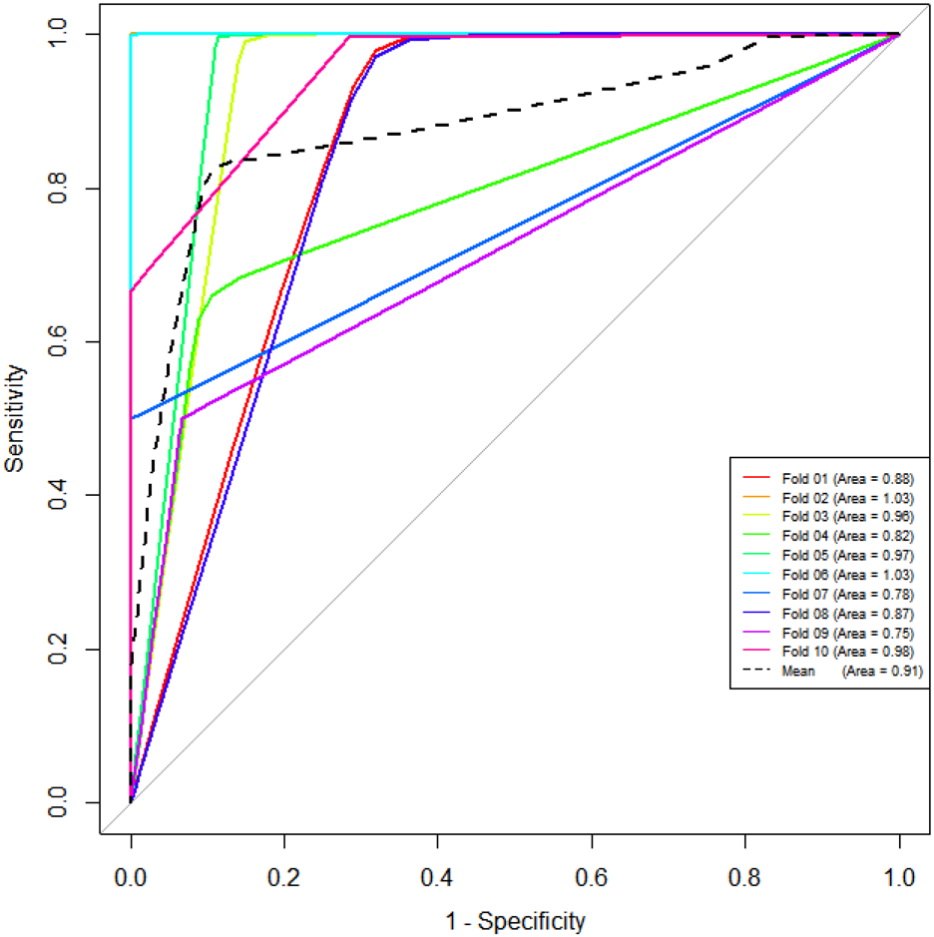
The tenfold cross-validation in the testing set was performed to verify the stability of radiomics-only logistic model. The mean AUC was 0.910, which was similar to the calculated 0.923 in the testing set.

To the radiomics-only logistic model, there were finally 5 optimal radiomics features extracted. The AUCs were 0.975 (95%CI 0.921–0.996) in the training set and 0.923 (95%CI 0.797–0.982) in the testing set. The tenfold cross-validation was performed in the testing set, the mean AUC was 0.910 (Fig. 1), which was similar to the calculated result above. It demonstrated the good stability of radiomics-only logistic model. To the whole cohort, the AUC of radiomics-only logistic model was 0.958 (95%CI 0910–0.985) (Table 2).

**Table 2:** The accuracy of differentiated logistic models.

Logistic model	AUC	95%CI
Radiomics-only (training set)	0.975	0.921–0.996
Radiomics-only (testing set)	0.923	0.797–0.982
Radiomics-only	0.958	0.910–0.985
Convention-radiomics CT nomogram	0.968	0.923–0.990

### Performance of convention-radiomics CT nomogram

**Figure 2:**
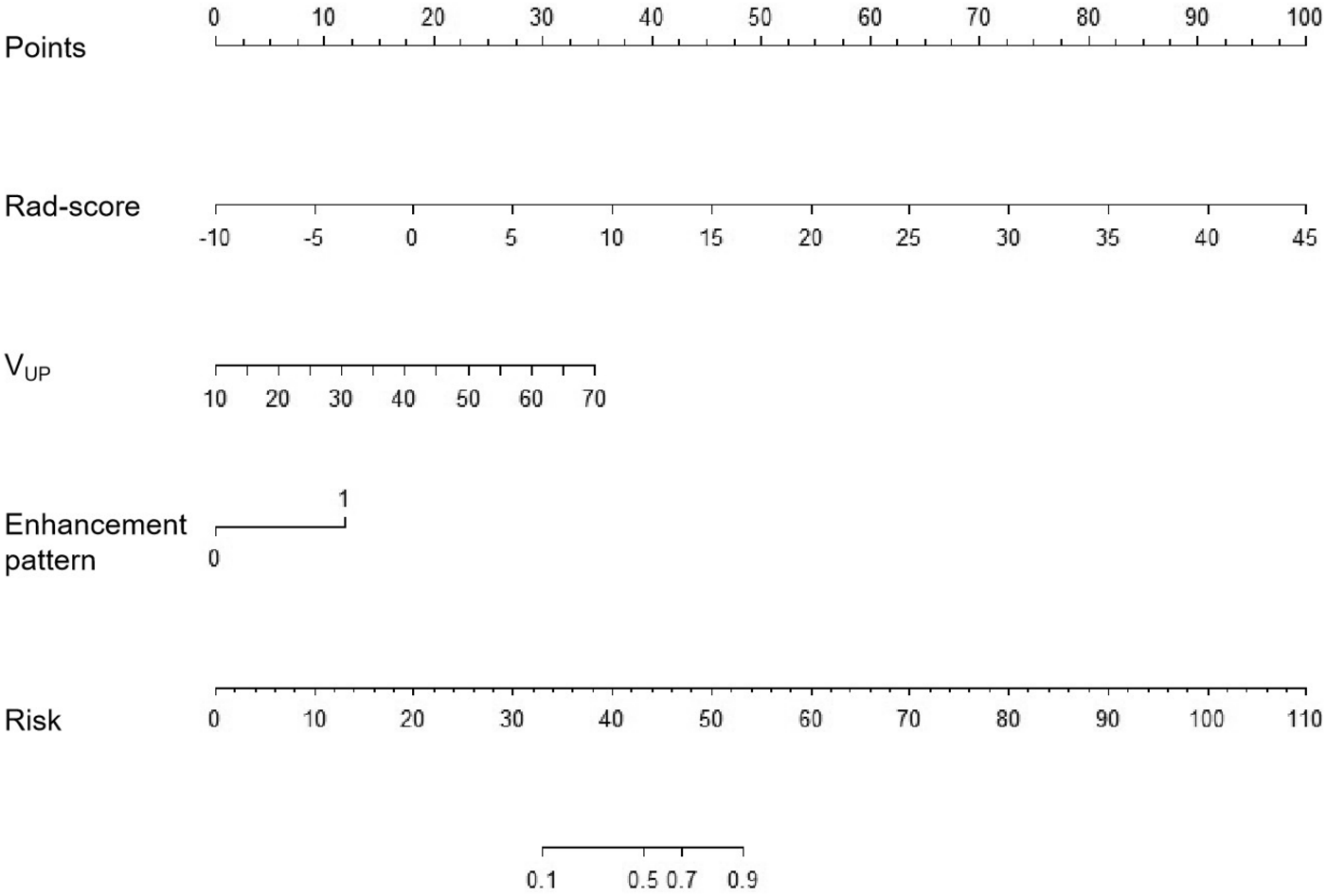
Convention-radiomics nomogram in differentiating fp-AML from ccRCC. The nomogram was constructed based on the whole cohort of 139 patients.

**Figure 3:**
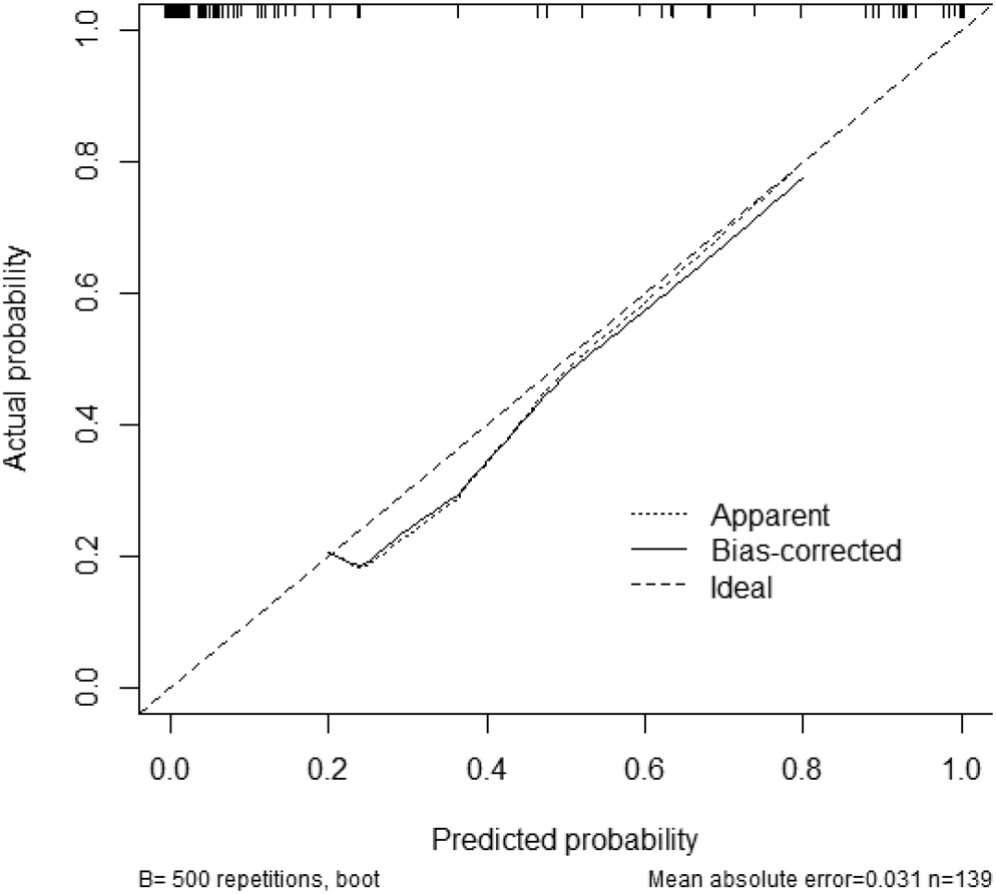
The calibration curve of the convention-radiomics CT nomogram.

**Figure 4:**
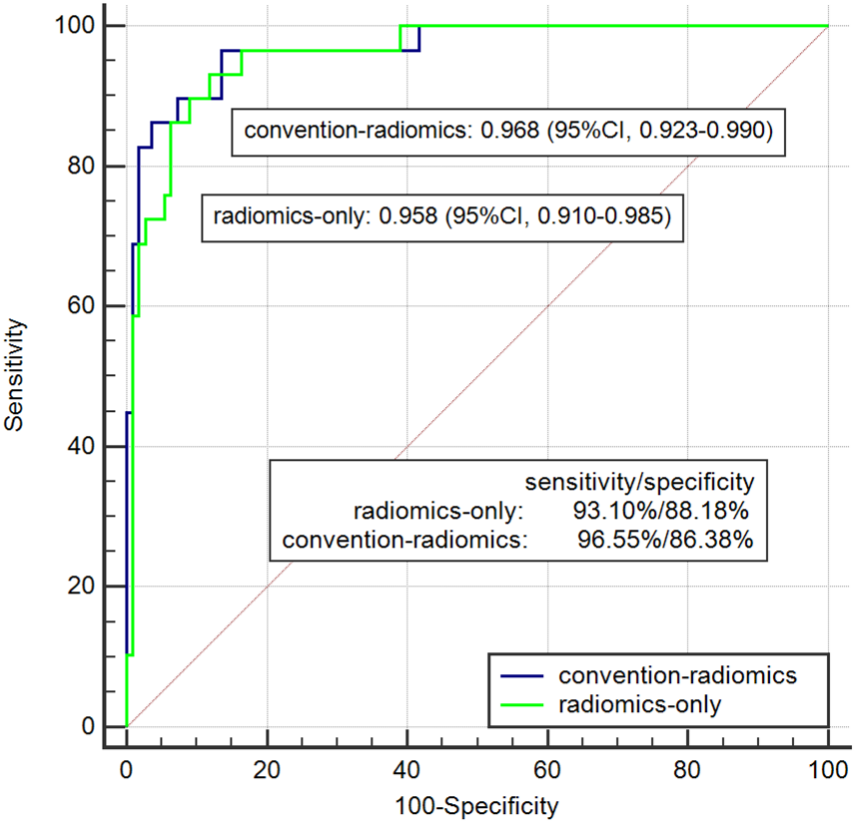
The comparison of logistic models of radiomics-only and convention-radiomics. The AUC of the convention-radiomics logistic model was higher than that of radiomics-only.

The convention-radiomics CT nomogram was constructed based on the whole cohort 139 patients including the training and testing set. After the multivariate logistic regression, the variables including enhancement pattern, V_UP_, and rad-score were identified as independent predictors for convention-radiomics logistic model. The rad-score of ccRCC was smaller than that of fp-AML. The convention-radiomics CT nomogram containing the three predictors was constructed (Fig. 2). The calibration curve of the convention-radiomics CT nomogram showed good calibration of the cohort (Fig. 3). The AUC of the convention-radiomics CT nomogram was 0.968 (95%CI 0.923–0.990) (Table 2). The ROC curves based on the whole cohort of the radiomics-only logistic model and convention-radiomics logistic model were plotted to compare the diagnosis accuracy. The convention-radiomics CT nomogram was superior to radiomics-only analysis in differentiating fp-AML from ccRCC (Fig. 4).

## Discussion

Conventional CT characteristics including pseudocapsule, cyst, enhancement pattern, V_UP_, R_CMP_%, and R_NP_% have been demonstrated to be significant in differentiation fp-AML and ccRCC. Previous studies have proved that ccRCC was easier to be heterogeneous, because of necrosis, calcification, or hemorrhage in lesions^18^. While fp-AML was more likely to be small size and to be heterogeneous in texture tendency than ccRCC^19^. The absence of pseudocapsule is another characteristic of fp-AML. There was a significant difference in pathologically confirmed pseudocapsule between fp-AML and ccRCC^20^. Though there was no statistic significance in the sign of calcification (*p* = 0.660). Inconsistent with our finding, AML rarely contains calcification, and its presence raises the possibility of RCC^21^. Previous studies have evaluated the enhancement pattern of fp-AML, typically showed early avid and washout over time in the corticomedullary and nephrographic phases^12^. While the most common enhancement of fp-AML was similar to ccRCC. See et al. found that V_UP_ of fp-AML was significantly different from that of ccRCC^19^. There was similarity in enhanced CT attenuating between ccRCC and fp-AML. Hence, the relative enhancement values, such as absolute washout ratio, enhancement change were induced to improve diagnostic accuracy and confidence^19^. This study standardized the CT attenuating by the aorta CT value, such as R_CMP_% and R_NP_%, to reduce the individual and technology variation. The relative CT ratio showed a statistical difference between fp-AML and ccRCC. While there is no specific CT characteristic that can accurately differentiate fp-AML from ccRCC.

Machine learning is useful in differentiating fp-AML from all RCC, ccRCC, and non-ccRCC, and it is free from the experience of radiologists during morphological interpretation^22^. Therefore further radiomics signature was analyzed in this study. The rad-score as a quantitative indicator for radiomics signature was calculated, and we found that the rad-score of fp-AML was larger than that of ccRCC. Our results highlighted the value of V_UP_, which was more sensitive than the V_CMP_ and V_NP_ in differentiating fp-AML from ccRCC. It was consistent with the conclusion from Lifen yan et al.^23^. They observed a trend toward better lesion distinction in UP for fp-AML versus ccRCC^23^. Zhichao feng et al. found that most optimal features were extracted from the UP and NP images^24^. Moreover, they found that the median and 75th percentile for UP and entropy for NP maybe potential quantitative imaging biomarkers for distinguishing fp-AML from ccRCC^24^. Taryn et al. evaluated the CT texture differentiation between fp-AML and RCC, and found that RCC was characterized by a lower degree of lesion homogeneity and a higher degree of gray-level entropy than fp-AML on UP CT^25^.

However, radiomics signature cannot replace the conventional CT analysis, as the former studies^25^ do not achieve 100% accuracy in differentiating, which is equal to our outcome. Therefore, a convention-radiomics CT nomogram provides a comprehensive method to simultaneously evaluate conventional CT characteristics, as well as to incorporate radiomics signature. The AUC of convention-radiomics CT nomogram was higher than that of radiomics-only analysis, elevating the distinguishing accuracy from 0.958 to 0.968. The enhancement pattern, V_UP_, and rad-score were independent predictors of the convention-radiomics CT nomogram. The lesions appeared heterogeneous enhancement pattern, with relative lower V_UP_ and smaller rad-score were inclined to be diagnosed as ccRCC. The nomogram is easier for radiologists to utilize in clinical routine, which could be performed quickly and get the clinical risk factors. Therefore, convention-radiomics CT nomogram could help better differentiate fp-AML from ccRCC.

There were several limitations to our study. Firstly, the unequal amount and single institutional patients limited the accuracy of differentiation. The multi-center and more diverse prospective study is needed. Secondly, this convention-radiomics nomogram lacked external validation. Thirdly, owing to the insufficient sample size, the two-group data did not achieve a complete match in size, gender, and age. Fourthly, because of the abandon of patients without surgeries. It gives rise to selection bias. Hence, larger-scale samples are essential to diminish the difference between groups.

In conclusion, the convention-radiomics CT nomogram exhibited favorable performance in differentiating fp-AML from ccRCC in CT images, compared with the radiomics-only analysis. The enhancement pattern, V_UP_, and rad-score were independent factors for distinguishing two diseases.

## Methods

### Patients selection

This retrospective study was approved by the ethics review board of Zhejiang Provincial People’s hospital. The ethics committee approved that this retrospective study can waive informed consent. The methods were performed by the relevant guidelines and regulations. All data were reviewed from 2013 to 2019 according to the following criteria: (1) with three-phase CT examination including unenhanced phase (UP), corticomedullary phase (CMP), and nephrographic phase (NP) based on the same CT protocols. (2) Histopathologically confirmed after partial or total resection surgeries. (3) Macroscopic fat was not been observed in lesions on UP CT images by two radiologists. (4) Solid dominant lesions, without a large proportion of necrotic fluid and hemorrhage.

Finally, there were 139 patients (29 fp-AML patients, 110 ccRCC patients) in this study. All patients were divided into the training set (97 patients, 20 fp-AML/77 ccRCC) and testing set (42patients, 9 fp-AML/33 ccRCC) with a proportion of 7:3, randomly.

Two radiologists with 7–10 years of abdominal diagnostic experience depicted the ROIs of lesions and assessed the conventional CT characteristics, respectively, and the intra-observer repeatability was evaluated by the intra-class correlation coefficient (ICC). We compared the data of six independent conventional CT risk factors and rad-score by two radiologists. The ICC of two radiologists was between 0.782 and 0.931, which greater than 0.75 was considered to be of good agreement. Eventually, the data from two radiologists with mutual consensus after discussion and adjudication was adopted.

### CT examination and ROI segmentation

CT examinations of recruited patients were performed by Smatom Definition AS 64/128 (Siemens Healthcare). By the technology of computer-assisted bolus-tracking, taking a 100Hu threshold in the abdominal aorta at the level of the celiac artery as the baseline, and the scan delays were respectively 15 s and 30 s for the CMP and NP. The antecubital vein of the patient was injected with 90–100 ml contrast material (iopromide, 370; Bayer) at a rate of 3.0 ml/s. The scanning parameters were as follow: tube current, 200 mA; tube voltage, 120kVp; rotation time, 0.75 s; detector collimation, 64*0.625 mm; pitch, 1.375; slice thickness and reconstruction thickness, 5 mm.

**Figure 5:**
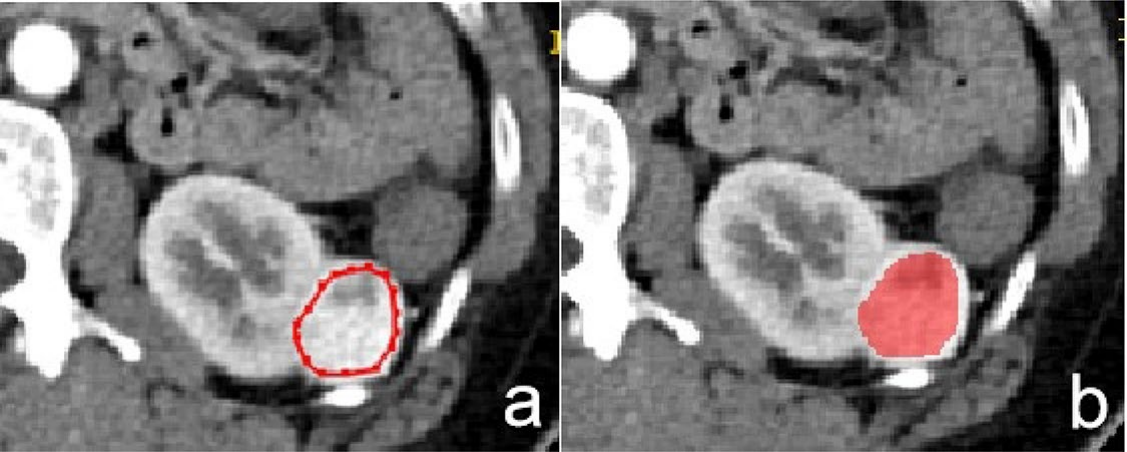
Manual defined smooth curve ROI was delineated, given 2–3 mm distance from the tumor margin.

The window width and window level of CT images were 300Hu and 40Hu, respectively. The smooth curve of whole-tumor ROI was delineated slightly smaller (2-3 mm from the actual lesion margin) in size, manually (Fig. 5a,b). The ROIs were delineated in “ITK-SNAP” (http://www.itksnap.org/; V 3.4.0 ).

### Conventional CT analysis

**Figure 6:**
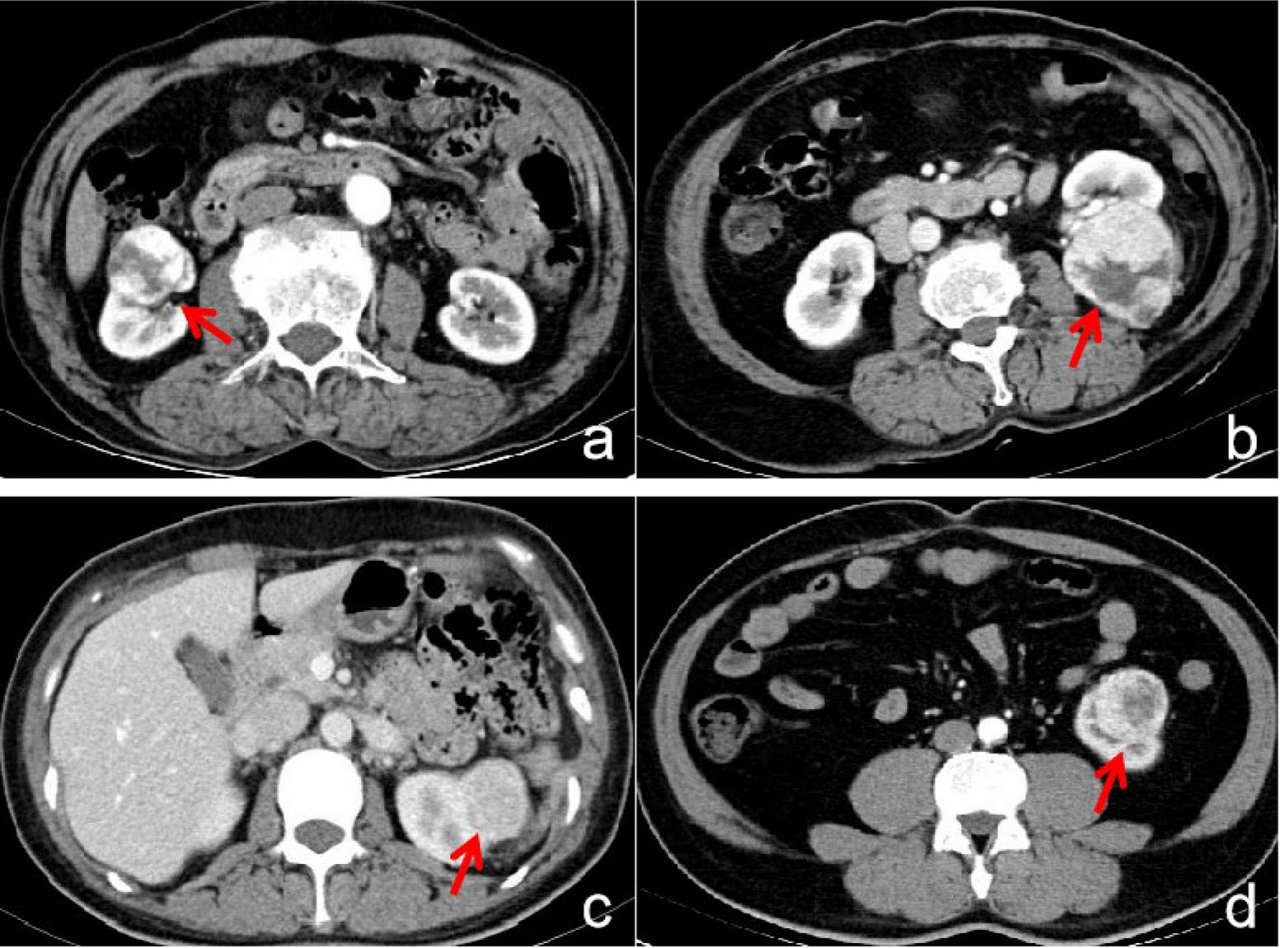
(**a**) A 52-year-old man with ccRCC, the CT image of CMP shows the sign of pseudocapsule (arrow). (**b**) A 51-year-old man with ccRCC, the CT image of NP shows cyst region in the mass (arrow). (**c**) A 39-year-old woman with fp-AML, the CT image of NP shows the homogeneous enhancement pattern (arrow). (**d**) A 55-year-old man with ccRCC, the CT image of CMP shows the heterogeneous enhancement pattern (arrow).

The conventional CT characteristics include qualitative and quantitative CT characteristics. The qualitative CT characteristics cover position(left/right), pseudocapsule, calcification, cyst, and enhancement pattern. The pseudocapsule surrounding the RCC is a fibrous tissue, displaying a low attenuating boundary between RCC and normal renal parenchyma on enhanced CT^26^ (Fig. 6a). The cyst is displayed as water density in CT images (Fig. 6b). The enhancement pattern is classified into homogeneous and heterogeneous^27^ (Fig. 6c,d). The quantitative CT characteristics encompass V_UP_, V_CMP_, V_NP_, R_CMP_%, and R_NP_%. V_UP_ = tumor_UP_, V_CMP_ = tumor_CMP_ − tumor_UP_, V_NP_ = tumor_NP_ − tumor_CMP_, R_CMP_% = V_CMP_/aorta_CMP_*100, and R_NP_% = V_NP_/aorta_NP_*100. The relative CT ratio normalized by aorta CT value, which is relative to individual variability and contrast material bolus could be more sensitive for distinguishing small renal lesions^28^.

### Radiomics signature analysis

The total 396 features are constituted of histogram parameters, texture parameters, form factor parameters, GLCM parameters, and RLM parameters. Before the analysis of radiomics signature, three preprocessing steps including re-sampling with 1.0 m at X/Y/Z-spacing, denoising by Gaussian, and discretizing the gray level from 0.0 to 255.0 by AK software (Artificial Intelligence Kit V 3.0.0, GE Healthcare) were taken, automatically. Then, it took three steps for feature selection: (1) replacing abnormal values by mean for standardization. (2) Automatically partitioning the training and testing set with a proportion of 7:3. (3) Through methods of ANOVA/MW, correlation analysis, and LASSO, features were further reduced from redundant dimensions. To verify the stability of radiomics signature model, tenfold cross-validation was performed in the testing set (Fig. 1).

The radiomics-only logistic model incorporated three-phase CT images were constructed to evaluate the predictive accuracy of radiomics signature in differentiation fp-AML from ccRCC. And the corresponding rad-score was calculated according to the following formula: rad-score = 4.85 + 1.60*[RMS] − 1.49*[ClusterShade_angle90_offset1] − 1.23*[ClusterShade_angle90_offset4] + 89.22*[Inertia_AllDirection_offset4_SD] − 1.33*[sumEntropy2]. The AUC was calculated to quantify the accuracy of radiomics signature in distinguishing two diseases.

### Construction of convention-radiomics CT nomogram

The univariate analysis was applied for conventional CT characteristics, including qualitative characteristics (position, pseudocapsule, calcification, cyst, and enhancement pattern) and quantitative characteristics (V_UP_, V_CMP_, V_NP_, R_CMP_%, and R_NP_%) to select independent conventional CT risk factors. Multivariate logistic regression combining the independent conventional CT risk factors with rad-score was performed to construct a convention-radiomics logistic model for differentiating fp-AML from ccRCC. A convention-radiomics CT nomogram was developed according to the relevant logistic model. A calibration curve was used to evaluate the calibration of the nomogram. The ROC curve was calculated to assess the distinguishing accuracy of convention-radiomics CT nomogram.

### Statistical analysis

The radiomics signature analysis and logistic model construction were completed with R software (V 3.6.1). The logistic models of radiomics-only and convention-radiomics were completed with “rms” package. And the rad-score was calculated. The univariate analysis containing the independent-samples T-test and Pearson chi-square test, and multivariate logistic regression were done with SPSS (IBM, V 22.0). The convention-radiomics CT nomogram and calibration plots were completed with “rms” package by R software. The ROC curves were delineated with MedCalc. The value of *p* < 0.05 was considered to have significance.

The intra-class correlation coefficient (ICC) was calculated to estimate the intra-observer agreement. Two radiologists with 7–10 years of abdominal diagnostic experience depicted the ROIs of lesions and assessed the conventional CT characteristics, respectively. We compared the data of six independent conventional CT risk factors and rad-score by two radiologists, the ICC greater than 0.75 prompted good agreement.

## Data Availability

The data sets generated during and/or analyzed during the current study are available from the corresponding author on reasonable request.
